# A Novel Range-Extended Strategy for Fuel Cell/Battery Electric Vehicles

**DOI:** 10.1155/2015/363094

**Published:** 2015-07-05

**Authors:** Jenn-Jiang Hwang, Jia-Sheng Hu, Chih-Hong Lin

**Affiliations:** ^1^Department of Greenergy, National University of Tainan, Tainan 700, Taiwan; ^2^Department of Mechanical Engineering, National Cheng Kung University, Tainan 701, Taiwan

## Abstract

The range-extended electric vehicle is proposed to improve the range anxiety drivers have of electric vehicles. Conventionally, a gasoline/diesel generator increases the range of an electric vehicle. Due to the zero-CO_2_ emission stipulations, utilizing fuel cells as generators raises concerns in society. This paper presents a novel charging strategy for fuel cell/battery electric vehicles. In comparison to the conventional switch control, a fuzzy control approach is employed to enhance the battery's state of charge (SOC). This approach improves the quick loss problem of the system's SOC and thus can achieve an extended driving range. Smooth steering experience and range extension are the main indexes for development of fuzzy rules, which are mainly based on the energy management in the urban driving model. Evaluation of the entire control system is performed by simulation, which demonstrates its effectiveness and feasibility.

## 1. Introduction

In order to reduce our dependence on petrochemical resources and decrease the deterioration of the earth's environment, the United Nations (UN) passed the “Kyoto Protocol” in December of 1997. Basically, the Kyoto Protocol progressively restricts the emissions of carbon dioxide (CO_2_) in every industrialized country. Consequently, this protocol has resulted in a massive impact on vehicle engineering. The result is a new challenge to the design of revolutionary vehicles for the future. Thus, the proposal of novel, low-pollution, and pollution-free vehicles has increased including concepts such as hybrid, ethanol, hydrogen fuel cell, and electric-power motor cars. In these next-generation automobiles, the fully electric motor vehicles have pollution-free benefits, which drive further research and development [1–3]. Compared to their conventional gasoline counterparts, these electric cars can achieve quieter and pollution-free operation. Electric cars are driven by electrical motors and are powered by batteries. Therefore, the prospects of the electric automobile industry are brightening. However, with respect to the driving experience, the electric vehicle has the following drawbacks.

- The battery charging requires ample time—several hours for common home chargers.
- Currently, the maximum range of a battery-powered car is less than its gasoline engine counterpart, which thus creates range anxiety.

In order to endow the electric vehicle with a similar driving experience as the gasoline engine car, the range-extended electric vehicle (REEV) [4, 5] is proposed. The range-extended electric vehicle is presented to mitigate the range anxiety of electric vehicles. Conventionally, the electric vehicle is range-extended by a gasoline/diesel generator. The generator charges the battery during times of rest. This idea can assist the battery's state of charge (SOC) in maintaining a higher level. Consequently, as the generator is in operation, the vehicle is not in a pollution-free mode, which, to the dismay of many, is therefore not compliant with the aim of zero-CO_2_ emission. The fuel cell range-extended electric vehicle (FC-REEV) is presented in response to these concerns. Unlike the hybrid vehicles, the fuel cell only acts as the energy supplier to the battery. The entire powertrain is in a cascade formation. Of course, if the fuel cell power is sufficient, a fuel cell generator can supply electricity to the battery and electric motor simultaneously, forming the conventional hybrid powertrain. Nevertheless, the fuel cell hybrid powertrain cannot have any benefit on fuel economy. Since the power of fuel cell is large enough to drive the electric motor, the relay battery becomes unnecessary. This type of vehicle is called the fuel cell electric vehicle (FC-EV), or simply the fuel cell vehicle. Unlike FC-EV, the FC-REEV is cascaded with a battery. It utilizes a smaller power fuel cell to charge the battery while it is being driven. This scheme can ensure a higher SOC on battery packs and achieves the range extension in consequence. This idea is very suitable for urban-life electric vehicles.

Conventional REEVs use the switch regulation to control the fuel cell generator for charging. Basically, the generator initiates charging when the SOC drops to less than 20% (or in some studies, 30%) [6]. If the SOC is lower than the warning level, the generator is activated to charge the battery. This action is terminated when the SOC reaches 80%. This approach surely can recover some SOC during steering. However, there are still some disadvantages.

- If the SOC is at a low level, the vehicle will fall into a stop-and-go period. Otherwise, the vehicle will stop for a long time, and the driver will have to wait for the SOC to recover.
- The battery cannot perform charging and discharging at the same time. In highway driving, due to the continuous pedal command requests, it is difficult for the generator to charge the battery. Consequently, the SOC will drop quickly under this scenario.

In order to solve these problems, Chen et al. [7] present a dynamic programming strategy based on a multimode switch control. This paper presents a novel charging strategy based on fuzzy turning rules for fuel cell/battery electric vehicles. It also can achieve similar performances. Fuzzy control has revealed its superior performance on electric vehicle applications [8]. Compared to the conventional switch control, a fuzzy control approach is employed in this study to improve the battery SOC. This methodology can resolve the quick SOC loss problem in the conventional approach and has the potential to eliminate range anxiety. The battery's lifetime and fuel economy are of concern in the development of fuzzy rules, which are mainly based on the energy management in the urban driving model.

This paper aims to make use of the advantages of electric vehicles driven by a fuel cell/battery to reveal a new concept on REEV. It is structured as follows. The system modeling is introduced in Section 2, followed by the fuzzy control strategy for the presented FC-REEV in Section 3.  Section 4 gives illustrated examples by simulation for evaluating the investigated strategy. Finally, Section 5 offers some concluding remarks.

## 2. Modeling

### 2.1. Fuel Cell Model

**Figure 1:**
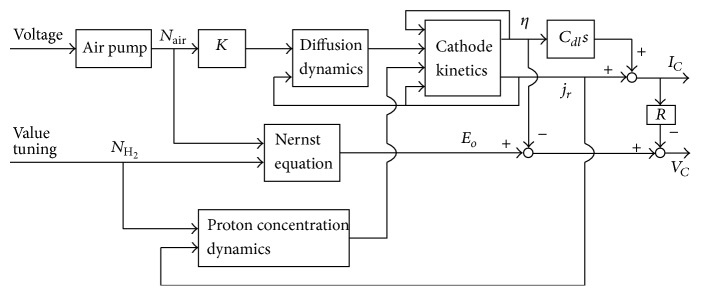
PEMFC dynamics.

The fuel cell system considered in the simulation study is based on the design manufactured by Asia Pacific Fuel Cell Technologies, Ltd. This system is powered by a proton exchange membrane fuel cell (PEMFC). The inputs of the system are hydrogen and air, while the outputs are cell voltage and current. The dynamics of the fuel cell system is nonlinear and time varying. It is influenced by many factors, including the diffusion dynamic, the Nernst equation, proton concentration dynamics, and cathode kinetics as illustrated in Figure 1 [9, 10]:

 diffusion equation:
  (1) $$\begin{array}{c} R_{ohm}=R_{ref}+\alpha_{T}\left(T-T_{ref}\right); \end{array}$$
 Nernst equation:
  (2) $$\begin{array}{c} E_{o}=E_{ref}+\frac{dE_{o}}{dT}\left(T-T_{ref}\right)+k\frac{RT}{zF}ln\left(P_{H_{2}}P_{O_{2}}^{0.5}\right); \end{array}$$
 proton concentration dynamics:
  (3) $$\begin{array}{c} u\left(\frac{-\partial C_{H^{+}}}{\partial t}\right)\frac{\partial C_{H^{+}}}{\partial t}+\frac{C_{H^{+}}}{\tau_{H^{+}}}=\frac{1+\alpha_{H^{+}}+j^{3}}{\tau_{H^{+}}}; \end{array}$$
 cathode kinetics:
  (4) $$\begin{array}{c} \eta =bln\left\{\frac{p_{10}{\left[H^{+}\right]}_{0}}{p_{1}\left[H^{+}\right]}\left(1+\frac{j_{r}}{j_{0}A_{r}}\right)\right\}. \end{array}$$

**Figure 2:**
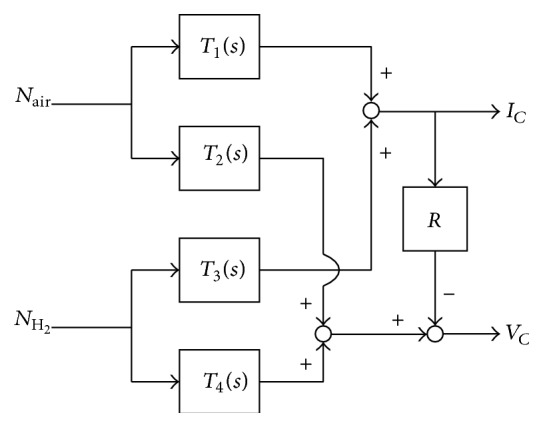
Block diagrams of fuel cell system.

From the system point of view, the physical model of Figure 1 can be represented as a multi-input and multioutput (MIMO) system, as depicted in Figure 2, with the following relation [11]:

(5) $$\begin{array}{c} I_{C}=T_{1}\left(s\right)N_{Air}+T_{3}\left(s\right)N_{H_{2}},\\ V_{C}=T_{2}\left(s\right)N_{Air}+T_{4}\left(s\right)N_{H_{2}}-RI_{C}. \end{array}$$

*T*_1_(*s*) ~ *T*_4_(*s*) represent the transfer functions of the subsystem. Note that the dynamics of linearized model (5) depends on the operating conditions.

### 2.2. Battery Model

**Figure 3:**
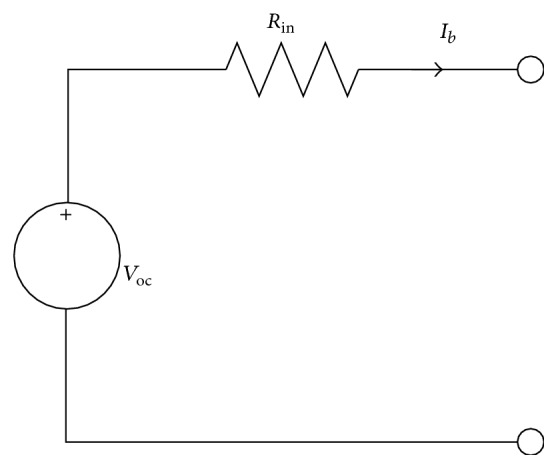
Equivalent circuit of simplified battery model.

The battery model in the simulation study is simplified as an equivalent circuit with a voltage source and a resistance [12–16], which is also called the *R*_int_ model and can be seen in Figure 3. The thermal effect on battery dynamics is ignored by assuming that the battery temperature will not experience major changes during the system testing. In Figure 3, *V*_oc_ is the open-circuit voltage of the battery, *R*_in_ stands for the internal resistance, and *I*_*b*_ represents the battery current. By considering the charging and discharging requirements, the SOC is obtained from the amount of capacity that remains after discharge from a top-of-charge condition as [17]

(6) $$\begin{array}{c} SOC\left(t\right)=Q_{T}-\overset{t}{\underset{t_{0}}{\int }}i\left(\tau \right)d\tau =Q_{T}-\Delta q. \end{array}$$

Herein, *Q*_*T*_ is the theoretical capacity of a battery and *i*(*τ*) is the current demand from electric motor. Note that Δ*q* can also be recovered when the fuel cell generator is charging the battery. Additionally, practical capacity *Q*_*P*_ of a battery is always much lower compared to the theoretical capacity *Q*_*T*_ due to practical limitations and battery protections. Herein,

(7) $$\begin{array}{c} Q_{P}\left(t\right)=\overset{t_{cut}}{\underset{t_{0}}{\int }}i\left(\tau \right)d\tau . \end{array}$$

Due to the fact that the behaviors of charging and discharging in chemical batteries are depending on the chemical reaction rate and safety control of battery protection, the current function *i*(*τ*) always falls on a nonlinear function. This behavior can be affected by room temperature, material fatigue, and so on. In this paper, the dynamics of employed battery is directly adopted from the simulation tools and will be revealed in Section 4 as well.

According to the investigation results in [16], obviously, to improve the dynamic voltage prediction precision of the battery model, recursive approaches are better due to the nonlinear behaviors of a battery. For example, the data-driven based parameters identification approach in [16] reveals a potential to achieve high voltage estimation accuracy against different aging levels and operation environments. Note that the behaviors to dynamic reactions on a chemical battery are sophisticated. Consequently, there are many battery models proposed to probe the system performance. Based on the similar testing setup in [15], the employed model (i.e., *R*_int_ model) reveals almost the same performance as the Thevenin Model, RC Model, PNGV Model, and DP Model. For some critical driving, the SOC prediction based on these approaches shows different errors. However, in urban driving cycles such as the NEDC, these approaches almost share the same reliability. In this paper, the driving scenarios are mainly based on the urban driving for range-extended fuel cell/battery electric vehicles. Consequently, the utilized battery model already has sufficient ability to carry out the analysis for the presented study.

### 2.3. Vehicle Dynamics

**Figure 4:**
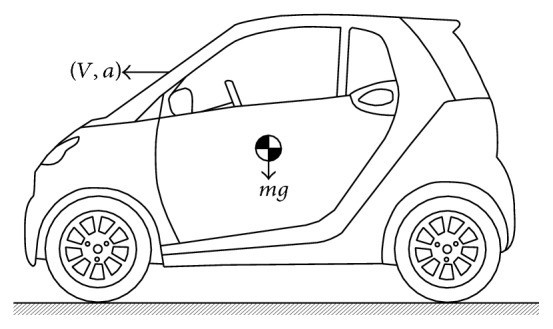
Vehicle model.

Consider a four-wheeled vehicle in a longitudinal motion, as depicted in Figure 4; the dynamic equations for the longitudinal motion of the vehicle can be found from Newton's second law of motion [18]. The forces of vehicle driving include air drag, rolling resistance, accelerating force, and climbing force. Hence, the power *P*_*d*_ needed in vehicle operation can be found as

(8) $$\begin{array}{c} P_{d}=\left(ma+C_{R}mg+mgsin\theta +\frac{1}{2}\rho_{a}C_{D}A_{F}V^{2}\right)V, \end{array}$$

where *m* is the mass of the vehicle, *a* stands for the acceleration of the vehicle, *C*_*R*_ is the coefficient of rolling resistance, *g* is the gravity constant, *θ* is the gradient angle, *ρ*_*a*_ is the density of air, *C*_*D*_ is the drag coefficient, and *A*_*F*_ is the frontal projected area of the vehicle. For regular steering, these mechanical power assumptions are the major sources of electric power supplied by a lithium-ion battery and fuel cell generator. Note that, in practice, the main battery also supplies electricity to consumptions such as air conditioning and headlights. In order to avoid missing the focus of analysis, these auxiliary power losses are omitted for simplification.

### 2.4. System Construction

**Figure 5:**
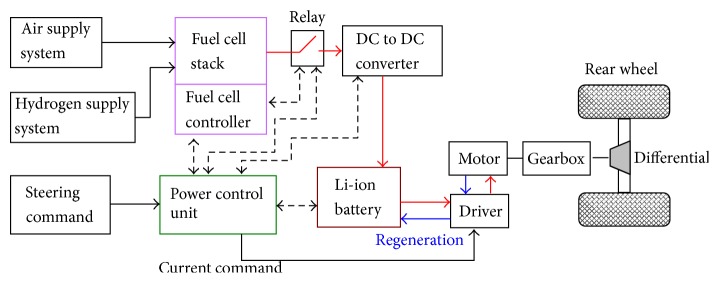
Schematics of presented FC-REEV system.

Figure 5 shows the schematics of the presented FC-REEV system. In order to evaluate the proposed range extension strategy, the whole system model, as shown in Figure 5, is implemented in the simulation of MATLAB/Simulink. As can be seen in this figure, a traction motor is employed for delivering torques to the rear wheels. The individual motor is a 10 kW three-phase AC 72V motor. The maximum motor speed is 2000 rpm. The fuel cell power is 4 kW, and the lithium-ion battery has a capacity of 105 Ah. The vehicle weighs 900 kg. Note that the motor can also be operated as a generator during the braking cycle. Basically, from the current direction, one can determine the operation mode. As depicted in Figure 5, a power control unit (PCU) plays an important role in the system management. In some studies, PCU is also referred to as the electronic control unit (ECU) because it regulates all of the subsystems. For example, the relay switch is controlled by PCU, which decides the activation of the fuel cell generator for battery charging. Ostensibly, different power management strategy results in different fuel economy and driving experiences.

## 3. Proposed Range Extension Strategy

### 3.1. Simulated Driving Cycle

**Figure 6:**
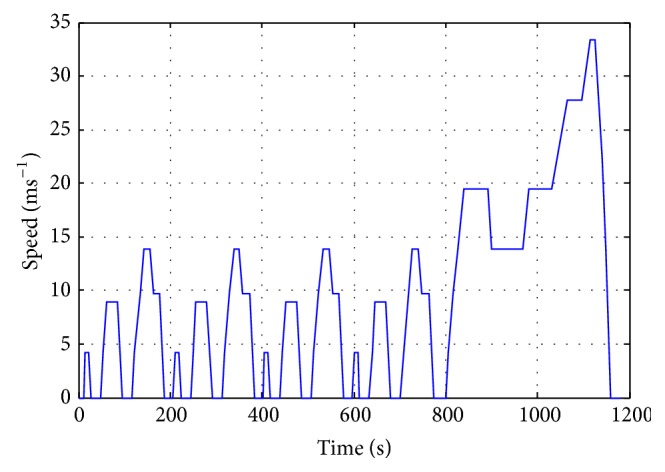
Driving pattern of NEDC.

Driving cycle is a simulation pattern for evaluating the vehicle's fuel economy in different scenarios. It is an important tool for design in driveline and control strategies. The main purpose in powertrain design is to minimize fuel consumption and component costs, while maximizing drivability. Since it is still cost-ineffective to build prototypes of FC-EV, the FC-REEV becomes an alternative in early stages of the development. The driving cycle is formed from numerous tests. Nowadays, it is a standard process in fuel economy evaluation. Basically, driving cycle is a speed profile where the most common scenarios of steering, such as rapid traction, braking, and coasting, are concerned. Its speed profile is defined as a function of time for a fair evaluation. Several driving cycles have been developed by governments around the world as tools for vehicle certification. The famous New European Drive Cycle (NEDC) in Europe and the Federal Test Procedure (FTP) in the USA are sufficient benchmarks. In this study, as shown in Figure 6, the driving pattern of NEDC is employed to evaluate the fuel economy and range extension. It is known that the NEDC is composed of four ECE-15 (urban driving cycle) segments as well as an EUDC (extraurban driving cycle) segment. Each ECE-15 cycle is 195 sec and 0.9941 km long, and its modeling covers the most typical urban driving scenarios in metropolitan areas. The EUDC is 400 sec and 6.9549 km long for high-speed urban driving. The combined fuel economy is calculated by a total consumption of urban and extraurban cycles over 10.9314 km. The entire test procedure requires 1180 sec with an average speed of 33.35 kmh^−1^.

In other words, one cycle of the NEDC is just 10.9314 km. It is not sufficient to represent all urban driving situations. Therefore, in this study, the driving pattern of NEDC is repeated ten times (i.e., 109.314 km) for a more realistic steering scenario. Under this assumption, the fuel economy and reliability of the FC-REEV can further reveal its major contribution to range extension.

### 3.2. Fuzzy Range Extension Strategy

As mentioned in the Introduction, because the battery cannot be charged and/or discharged simultaneously, the extended range of REEV is mainly relevant to the charging style to the battery. The conventional switch control strategy, which is also named the thermostat control strategy (TCS) in [19, 20], has achieved some success on REEV range extension; however, the battery charging dynamics is not nimble. It shows a drawback on a stop-and-go duration when the SOC is under a low level. The results give a clue that the generator power management could be more effective. In order to improve this inconvenient driving experience, this paper proposes a new range extension strategy based on fuzzy control.

**Figure 7:**
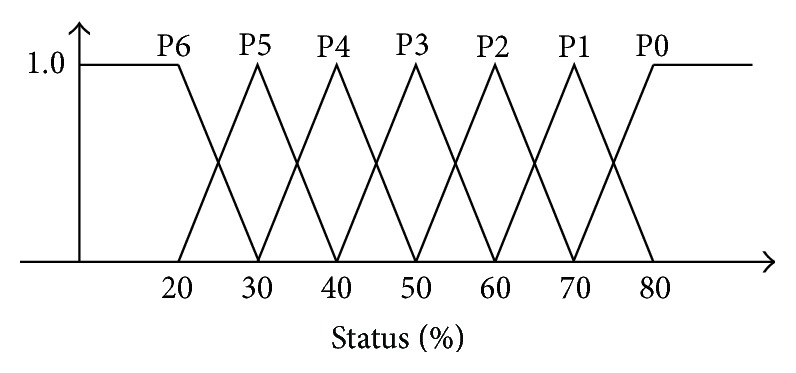
Triangular membership functions.

Generally, when controlling a process, human operators usually encounter complex patterns of qualitative conditions, which are not easy to quantify. For example, in many applications, the measurement data can be classified as fast, slow, high, low, and so on. Such linguistic variables are employed in describing inexact information. To represent such information, a new mathematical approach called fuzzy theory was proposed by Zadeh [21]. In essence, the fuzzy control is a mechanism that offers the designer to create solutions for control issues. It mimics and duplicates the techniques and knowledge based on human intelligence and decisions. In this paper, the fuzzy control is utilized for monitoring the battery's SOC. As illustrated in Figure 7, a fuzzy membership function (fuzzy sets) of the required status is defined with the following linguistic fuzzy set, where

 P0: status > 80%,
 P1: status = 70%,
 P2: status = 60%,
 P3: status = 50%,
 P4: status = 40%,
 P5: status = 30%,
 P6: status ≤ 20%.

The shape of the membership functions is quite arbitrary and is dependent on the user's preference. For the sake of mathematical simplicity, the triangular shape is utilized due to practical considerations. Note that there are many membership functions that can be utilized. The triangular membership function is one of the candidates. Based on the research results from Pedrycz [22], the triangular membership function works well in most of the industrial applications. Additionally, based on its linear and easier computation features, this study adopts this type of membership function to carry out the fuzzification process.

**Figure 8:**
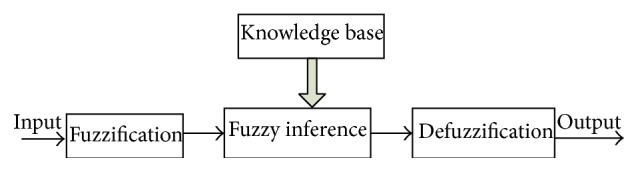
Fuzzy controller block diagram.

Due to the guarantee of expert experience, most commercial fuzzy products are rule-based systems. They receive the current states in the feedback loop and check the rules for control and operation. A basic fuzzy logic system can be found in Figure 8. Crisp input states are converted into fuzzy values based on fuzzy sets with the fuzzification block. The fuzzy controller is mainly based on the knowledge database denoted in the fuzzy rules. The decision-making-logic determines how the fuzzy logic operations are performed and precedes the outputs of each fuzzy rule in an “IF-THEN” policy. Finally, these processes are then converted into crispy values with the defuzzification block. The output crisp values then achieve the regulation for specific tasks.

Table 1 shows the presented fuzzy rules. Basically, these rules are derived from the experts' experience. The major rules consist of the following strategies.

*Case  1.* The fuel cell generator supplies 100% for battery charging. The pedal uses 0% for the acceleration. 

*Case  2.* The fuel cell generator supplies 67% for battery charging. The pedal takes 33% for the acceleration. 

*Case  3.* The fuel cell generator supplies 33% for battery charging. The pedal utilizes 67% for the acceleration. 

*Case  4.* The fuel cell generator supplies 44% for battery charging. The pedal uses up 56% for the acceleration. 

*Case  5.* The fuel cell generator supplies 22% for battery charging. The pedal receives 78% for the acceleration. 

*Case  6.* The fuel cell generator supplies 11% for battery charging. The pedal is allocated 89% for the acceleration. 

Note that the percentage of the charging/pedal sharing ratio is adjustable according to a specific index. Additionally, the distance is strictly indexed by ten cycles of NEDC (i.e., 109.314 km). The fuzzy controller receives statuses of the range and SOC from feedback sensors and regulates the duty ratio between the SOC and pedal.

## 4. Results and Discussions

**Figure 9:**
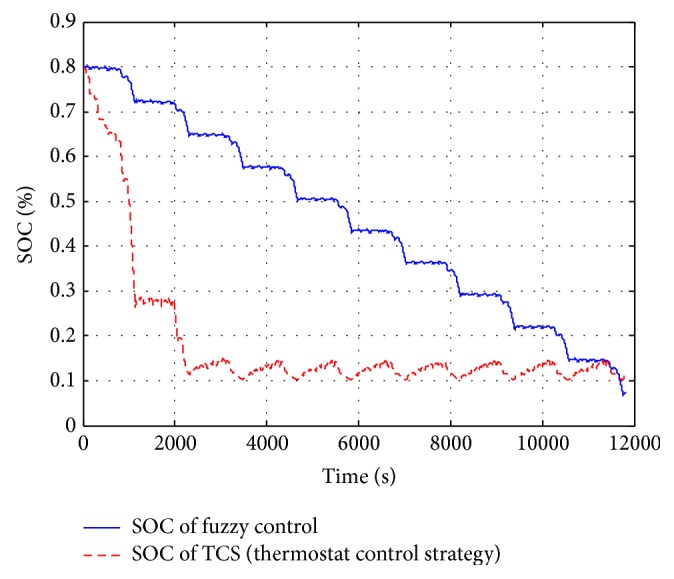
Comparative simulations of SOC.

In this section, the presented fuzzy control approach is tested in simulation for performance verification. The whole system of Figure 5 was employed as a platform for evaluation.  Figure 9 reveals the comparative results on SOC. As can be seen in this figure, the TCS approach falls into a stop-and-go status quickly. Conversely, the presented approach can reach the goal without any extra stops. Although the vehicle completes the test of range extension, the whole driving experience is very different. For the conventional approach, range anxiety still exists. The comparative simulation results confirm that the charging strategy plays an important role in the driving experience. From the viewpoint of range extension, both approaches achieve the goal. However, the steering experience is quite different; the fuzzy charging policy can maintain the SOC at a relatively high level to avoid the vehicle falling into a stop-and-go stage. The vehicle can accomplish nearly a no parking situation for battery charging. Fewer stops mean the vehicle's motion can follow the driving cycle smoothly and the kinetic energy can be sufficiently kept; hence, this finding represents a higher energy efficiency. In addition, the user does not feel significant change when driving with the proposed FC-REEV. Therefore, the driving experience of the proposed system will be almost the same as the conventional engine car.

**Figure 10:**
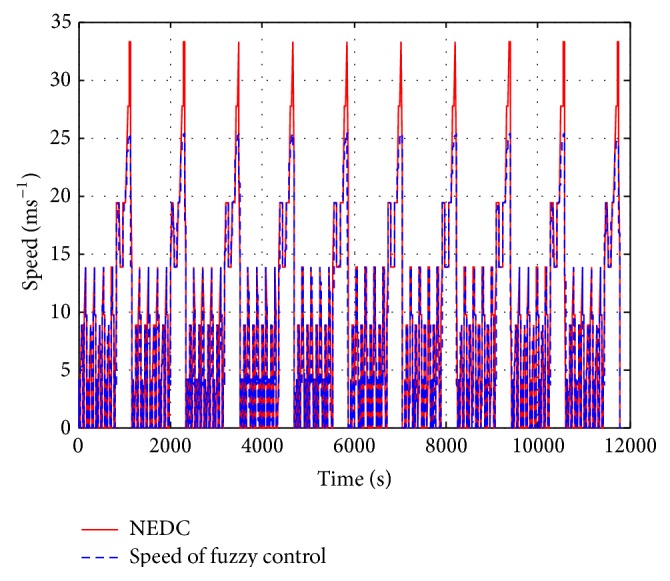
Speed profile of fuzzy control.

Figure 10 illustrates the speed performance of the proposed fuzzy control system. As can be seen in this figure, due to the duty management of pedal command sharing, the speed incensement will become sluggish in the high-speed zone. Conventionally, since the pedal command consists of the energy demand from the battery, there is no time slot for the battery to capitalize on charging from the generator. Moreover, because the battery in this scenario is continuously discharging, the SOC drops quickly as a result. In order to overcome the lack of time for charging the REEV during high-speed steering, the proposed fuzzy rules use the pedal time sharing concept to improve the quick loss problem. This idea utilizes the duty-sharing concept to gain some charging time during operation. This methodology solves the quick SOC loss problem and can maintain a smooth driving experience while the vehicle is at a high speed. The tradeoff is that the vehicle's speed increases slowly when the acceleration demand is suddenly given. However, due to the inertial momentum of vehicle, the motion behavior can be continuous. Hence, the proposed approach can still maintain safe steering for the operator and passenger(s). This experience is a better option than its stop-and-go alternative. Regarding the protection issue of a chemical battery's lifetime, a small discharge cycle is preferred than a long one [22]. Clearly, the presented fuzzy control attempts to avoid continuous deep discharge of the battery. This policy also facilitates the lifetime protection of battery packs. On the other hand, the conventional approach charges the battery when it is at a low SOC level. Multiple deep discharge cycles at this level will result in a fast deterioration and material decay of battery packs.

**Figure 11:**
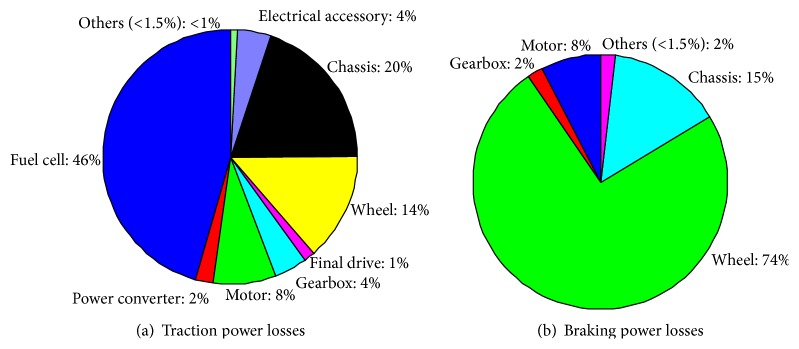
Vehicle traction/braking power losses.

Considering the fuel efficiency issue, Figure 11 shows the total power losses for the vehicle's traction and braking in the whole 109.314 km. The energy in fuel is consumed by various losses, such as the driving resistances, gear wearing, propelling kinetic energy, generator, and vehicular electronics. Basically, the fuel economy of a vehicle's propulsion is the fuel efficiency relationship between the traveled distance and the amount of energy consumed by the system. Additionally, different driving cycles and driver behaviors result in different outcomes. Maximizing the usages of chemical energy in fuel as much as possible on the propelling of the vehicle becomes a key task. As can be seen in Figure 11, the power loss during forward traction and braking is 15.1996 kW and 2.0481 kW, respectively. Obviously, the fuel cell generator dissipates the major power when the vehicle is in a traction state. Under the framework of system limitation, such as the structure of Figure 5, the only way to foster better fuel economy is to improve the energy efficiency of the generator. According to Demirdöven and Deutch [23], the PEMFC for transportation applications has higher energy efficiency than the conventional gasoline/diesel generator. Consequently, for REEV, the fuel cell generator is a suitable choice based on the inspection of fuel economy. Conversely, as the vehicle initiates braking, the major power is consumed by the wheel wearing. It is easy to realize that the energy losses from braking mainly come from the tires. The tires consume the majority of kinetic energy and hence stop the vehicle. If the motor driver can perform regeneration during braking, undoubtedly, the kinetic energy can be further recycled to achieve higher energy efficiency.

## 5. Conclusions

This paper has investigated the novel range extension strategy for fuel cell/battery electric vehicles. The analysis was carried out by the simulation results done by MATLAB/Simulink. The presented charging sharing idea, which is regulated by a fuzzy rule table, has revealed that the quick loss of SOC can be remedied for high-speed steering. The battery's lifetime and system's fuel economy have confirmed an improvement under the proposed energy management. These evaluations have demonstrated its effectiveness and potential feasibility. The main findings from the simulation results are described below.

1. Under the regulation of sufficient fuzzy control, the urban driving experience for FC-REEV can be the same as the internal combustion engine car. Hence, range anxiety can be fully managed in the proposed approach.
2. Fuzzy strategy also improves the battery's lifetime and fuel economy of a REEV system. A safe and continuous driving experience is guaranteed in the proposed FC-REEV. The operator can enjoy almost the same driving experience as the conventional engine car.
3. FC-REEV has revealed a better fuel economy than the conventional REEV. Additionally, the FC-REEV is a purely zero-CO_2_ emission car.
4. The energy consumption from the gearbox can be further saved when the powertrain design is changed. For example, the power decentralized electric vehicles that utilize the in-wheel motor to propel the vehicle's motion can achieve less heat loss on gear wearing. Investigations of relevant issues are worth studying in future work.

## Figures and Tables

**Table 1:** Fuzzy rules.

	P6 of distance	P5 of distance	P4 of distance	P3 of distance	P2 of distance	P1 of distance	P0 of distance

P6 of SOC	Case 1	Case 1	Case 2	Case 2	Case 2	Case 3	Case 3
P5 of SOC	Case 1	Case 1	Case 2	Case 2	Case 2	Case 3	Case 3
P4 of SOC	Case 1	Case 1	Case 2	Case 2	Case 2	Case 3	Case 3
P3 of SOC	Case 2	Case 2	Case 4	Case 4	Case 4	Case 5	Case 5
P2 of SOC	Case 2	Case 2	Case 4	Case 4	Case 4	Case 5	Case 5
P1 of SOC	Case 3	Case 3	Case 5	Case 5	Case 5	Case 6	Case 6
P0 of SOC	Case 3	Case 3	Case 5	Case 5	Case 5	Case 6	Case 6
